# Artificial Intelligence-Assisted Echocardiographic Image-Analysis for the Diagnosis of Fetal Congenital Heart Disease: A Systematic Review and Meta-Analysis

**DOI:** 10.31083/RCM28060

**Published:** 2025-04-27

**Authors:** Yaduan Gan, Lin Yang, Jianmei Liao

**Affiliations:** ^1^Department of Ultrasound, Zhangzhou Affiliated Hospital of Fujian Medical University, 363000 Zhangzhou, Fujian, China

**Keywords:** artificial intelligence, congenital heart disease, fetal echocardiography, diagnostic accuracy, meta-analysis

## Abstract

**Background:**

To assess the precision of artificial intelligence (AI) in aiding the diagnostic process of congenital heart disease (CHD).

**Methods:**

PubMed, Embase, Cochrane, and Web of Science databases were searched for clinical studies published in English up to March 2024. Studies using AI-assisted ultrasound for diagnosing CHD were included. To evaluate the quality of the studies included in the analysis, the Quality Assessment Tool for Diagnostic Accuracy Studies-2 scale was employed. The overall accuracy of AI-assisted imaging in the diagnosis of CHD was determined using Stata15.0 software. Subgroup analyses were conducted based on region and model architecture.

**Results:**

The analysis encompassed a total of 7 studies, yielding 19 datasets. The combined sensitivity was 0.93 (95% confidence interval (CI): 0.88–0.96), and the specificity was 0.93 (95% CI: 0.88–0.96). The positive likelihood ratio was calculated as 13.0 (95% CI: 7.7–21.9), and the negative likelihood ratio was 0.08 (95% CI: 0.04–0.13). The diagnostic odds ratio was 171 (95% CI: 62–472). The summary receiver operating characteristic (SROC) curve analysis revealed an area under the curve of 0.98 (95% CI: 0.96–0.99). Subgroup analysis found that the ResNet and DenNet architecture models had better diagnostic performance than other models.

**Conclusions:**

AI demonstrates considerable value in aiding the diagnostic process of CHD. However, further prospective studies are required to establish its utility in real-world clinical practice.

**The PROSPERO registration:**

CRD42024540525, https://www.crd.york.ac.uk/prospero/display_record.php?RecordID=540525.

## 1. Introduction

Congenital heart disease (CHD) is the most common congenital anomaly, affecting approximately 0.8% of the general population [1]. Despite significant advancements in diagnosis and treatment, CHD remains a common cause of infant mortality in the first year of life, accounting for approximately 30% of deaths due to congenital malformations [2, 3]. Early diagnosis and timely intervention have been shown to improve postnatal outcomes [4, 5]. Therefore, distinguishing normal fetal hearts from CHD is important. Fetal heart assessment is the primary method for detecting fetal CHD [6]. However, ultrasonographers still face significant challenges in obtaining high-standard, high-quality fetal heart images as required by guidelines due to ultrasound imaging artifacts, speckle noise, changes in fetal position and scanning angle, blurred image boundaries, and variations in the quality of images [7]. Although routine mid-pregnancy fetal heart ultrasound screening using the five heart views recommended by guidelines can detect approximately 90% of complex CHD [8, 9], in practice, the detection rate of CHD is only 30%–50%, with a sensitivity of approximately 40%–50% [10, 11], and largely depends on the personal experience of the ultrasonographer [12, 13]. Additionally, there are significant differences in the identification of normal fetal hearts and detection of abnormalities in health care systems amongst various regions [8, 14].

In recent years, a computer-aided approach that assists fetal operators in automatically identifying and interpreting the anatomical structures of the fetal heart has gained significant interest to address these challenges [15, 16]. Artificial intelligence (AI) methods, exemplified by deep learning (DL) [17], have found extensive applications in the field of medical image analysis, including tasks such as image classification, recognition, segmentation, registration, and computer-aided diagnosis. There is a computer-based method for fetal echocardiography. DL has found its most significant application in fetal ultrasound for pre-diagnostic purposes, such as detecting standard planes [18], classifying and identifying CHD [19, 20, 21], and evaluating the development of the fetal heart [22]. Studies have used AI in fetal echocardiography to improve the diagnostic accuracy of fetal CHD [23] and have shown that some AI models equal the performance level of experts [24, 25]. Recently, Arnaout *et al*. [26] retrospectively collected 107,823 ultrasound images from 1326 mid-pregnancy fetal screening cardiac videos and trained a neural network ensemble using the five views recommended in guidelines, including the three-vessel trachea view, three-vessel view, left ventricular outflow tract view, right ventricular outflow tract view, and abdominal view, to identify normal fetal hearts and CHD. The findings demonstrated that on an internal test dataset of 4108 fetal examinations, the model exhibited an area under the curve of 0.99 in distinguishing normal heart conditions from CHD. Its sensitivity reached 95%, while the specificity was 96%. Notably, the negative predictive value attained was 100%, accurately identifying cases without CHD. Gong *et al*. [20] proposed a new DGACNN model for the identification of fetal CHD by training 2655 normal hearts and 541 CHD cases. The model demonstrated good performance in identifying CHD, with an accuracy of 84%, surpassing cardiac experts. Additionally, the model addressed the problem of insufficient training datasets to train a stable model. Deep learning, the most advanced type of machine learning, has been applied to adult echocardiography and has been shown to outperform clinicians in judging images when the images are too small or have poor resolution.

Currently, there are several clinical studies on AI for the diagnosis of CHD. However, these studies are mainly single-center studies with small sample sizes and are limited to clinical research, with no available relevant evidence-based medicine. Therefore, this study aims to retrieve published relevant literature through systematic review and meta-analysis methods, scientifically synthesize and analyze the data, and obtain comprehensive and reliable evidence-based medicine evidence to comprehensively evaluate the accuracy of AI in diagnosing CHD.

## 2. Methods

### 2.1 Literature Retrieval and Inclusion

To gather relevant literature, a comprehensive search was performed across multiple databases, including PubMed, Embase, Cochrane, and Web of Science. The search focused on identifying English-language clinical studies published up until March 2024. This broad search strategy aimed to capture a comprehensive set of publications. In the PubMed database, we formulated our search approach using a combination of keywords and Medical Subject Headings (MeSH) terms originating from four essential concepts. The four key concepts included: artificial intelligence, imaging, fetal, and congenital heart disease. The specific search strategy is shown in **Supplementary Table 1**. All included studies had to meet the following criteria: (1) the researchers reported the values of true positives (TP), false positives (FP), true negatives (TN), and false negatives (FN) for the AI diagnosis of CHD, or values that could be calculated based on sensitivity (Se) and specificity (Sp); (2) the AI model was able to distinguish normal fetal hearts from CHD. Studies irrelevant to the topic, unpublished studies, case reports, abstracts, conference abstracts, non-English articles, and literature without complete diagnostic four-grid table data were not included. The current meta-analysis study was registered in the International Prospective Register of Systematic Reviews (PROSPERO) online database, under the registration number CRD42024540525.

### 2.2 Literature Screening and Data Extraction

Two reviewers independently conducted the literature screening and data extraction process after eliminating duplicate publications using EndNoteX9 (Clarivate, Philadelphia, PA, USA). First, titles and abstracts were read for initial screening to exclude literature unrelated to AI-assisted ultrasound diagnosis of CHD, reviews, or case reports. Then, the full text was read to exclude literature with incomplete information. Any discrepancies arising between the two reviewers were settled through deliberation or, when needed, resolved by a third reviewer’s adjudication. For the finally included articles, the full text and relevant references were thoroughly reviewed. A data extraction table was used to extract information, including: (1) basic information of the included studies, such as study title, corresponding author or first author, publication year, and study region; (2) study design type, reference standard, and type of CHD studied; (3) the AI model used in the study, with separate extraction of diagnostic accuracy for multiple AI models in one study. If a model’s dataset was validated multiple times, the highest Se and Sp were selected; (4) study outcome indicators, including basic information such as the number of positive results, TP, FP, FN, TN, as well as concordance rate, sensitivity, specificity, diagnostic odds ratio, positive likelihood ratio, negative likelihood ratio.

### 2.3 Quality Assessment of Included Studies

Adhering to the Cochrane Handbook guidelines, the quality assessment was conducted utilizing the quality assessment of diagnostic accuracy studies (QUADAS-2) tool. Subsequently, the Review Manager 5.4.1 software (Version 5.4.1, The Cochrane Collaboration, The Nordic Cochrane Centre, Copenhagen, Denmark) was employed to present the final evaluation findings in a comprehensive manner [27]. Literature quality assessment was conducted by two reviewers independently, and disagreements were resolved by discussion or adjudicated by a third author. Risk of bias and clinical applicability were evaluated by the QUADAS-2 tool. Patient selection, index test, reference standard, and flow and timing were used to assess risk of bias. Patient selection, index test, and reference standard were used to assess clinical applicability questions. Each question has three answer options, “yes/no/unclear”, for risk of bias. If the answers are “yes”, then the risk of bias is considered low. If the answers is “no”, there is a possibility of bias. There are no signaling questions for clinical applicability, only an overall assessment, with answer options including “high risk/low risk/unclear”. The “unclear” option could only be selected when the information provided in the literature was incomplete during the assessment process.

### 2.4 Data Analysis

The Stata15.0 software (Version 15.0, StataCorp, College Station, TX, USA) was used for statistical analyses. Calculations were performed to determine the pooled sensitivity, pooled specificity, positive likelihood ratio, negative likelihood ratio, and diagnostic odds ratio. The summary receiver operating characteristic (SROC) curve was plotted, and the corresponding area under the curve (AUC) value was computed. To assess potential publication bias, Deek’s test was employed as part of the analysis process. A *p* < 0.05 was considered to indicate publication bias in the included literature. We performed subgroup analyses based on the region and AI model to evaluate the individual performance of each index test and compare it with the diagnostic accuracy of all the combined modalities.

## 3. Results

### 3.1 Literature Screening Results and Process

**Fig. 1.**
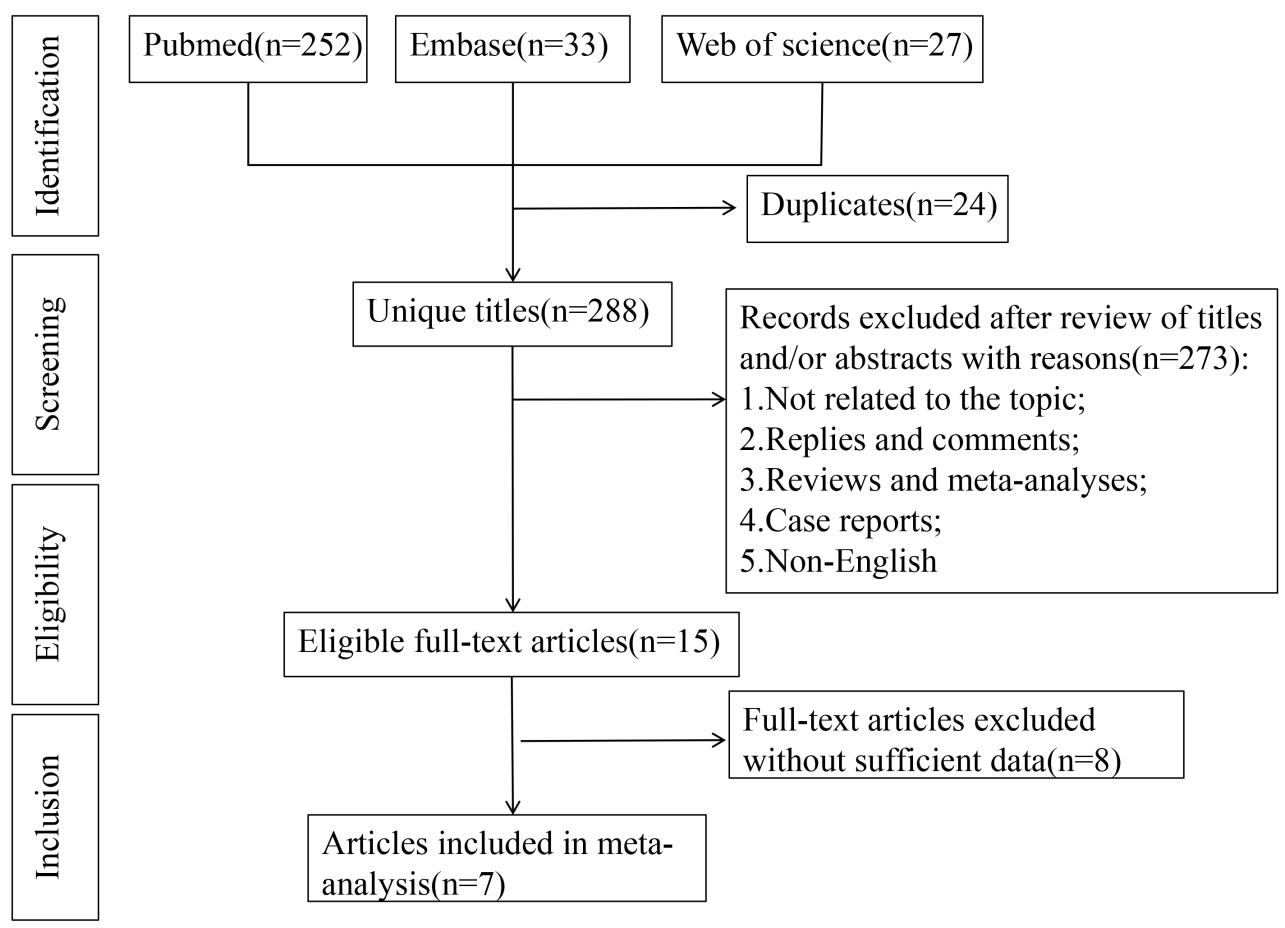
**The flowchart of the literature screening process**.

A total of 312 articles were obtained. After removing duplicates using EndNote, 288 articles remained. After reading titles and abstracts, 273 articles were excluded, resulting in 15 articles for initial screening. Among the 15 articles, 8 articles were excluded after reading the full text due to incomplete information and not meeting the requirements. The study ultimately included a total of 7 articles. The database format in the articles used grayscale images or selected frames from retained videos. Each frame required a uniform pixel. Fig. 1 illustrates the flowchart of the literature screening process, while Table 1 (Ref. [23, 26, 28, 29, 30, 31, 32]) presents the fundamental characteristics of the studies included in the analysis. If there were multiple models in the same study, they were extracted separately and distinguished by adding the letters “abcd” after the author.

**Table 1.** **Fundamental characteristics of the studies included**.

Authors	Study period	Country	Study design	Sample size	Type of CHD	Models
				positive/negative		
Wang *et al*. a [28]	2012–2022	China	retrospective	19/82	TAPVC	DeepLabv3+
Wang *et al*. b [28]	2012–2022	China	retrospective	19/82	TAPVC	FastFCN
Wang *et al*. c [28]	2012–2022	China	retrospective	19/82	TAPVC	PSPNet
Wang *et al*. d [28]	2012–2022	China	retrospective	19/82	TAPVC	DenseASPP
Athalye *et al*. [29]	2015–2016	Netherlands	retrospective	66/44	CHD	DL
Nurmaini *et al*. a [30]	2021	Indonesia	retrospective	952/177	CHD	DenseNet201
Nurmaini *et al*. b [30]	2021	Indonesia	retrospective	952/177	CHD	DenseNet121
Nurmaini *et al*. c [30]	2021	Indonesia	retrospective	952/177	CHD	ResNet50
Nurmaini *et al*. d [30]	2021	Indonesia	retrospective	952/177	CHD	ResNet101
Arnaout *et al*. a [26]	2000–2019	USA	retrospective	37/88	CHD	Ensemble
Arnaout *et al*. b [26]	2000–2019	USA	retrospective	37/4071	CHD	Ensemble
Day *et al*. a [23]	NA	UK	retrospective	250/250	AVSD	ResNet50
Day *et al*. b [23]	NA	UK	retrospective	250/250	AVSD	ResNet50
Day *et al*. c [23]	NA	UK	retrospective	250/250	AVSD	ResNet50
Day *et al*. a [31]	2014–2019	UK	retrospective	3960/6288	HLHS	ResNet50
Day *et al*. b [31]	2014–2019	UK	retrospective	59/102	HLHS	ResNet50
Day *et al*. c [31]	2014–2019	UK	retrospective	59/102	HLHS	ResNet50
Taksøe-Vester *et al*. a [32]	2008–2018	Denmark	retrospective	73/7300	COA	U-Net
Taksøe-Vester *et al*. b [32]	2008–2018	Denmark	retrospective	73/7300	COA	U-Net

HLHS, hypoplastic left heart syndrome; TAPVC, total anomalous pulmonary venous connection; AVSD, atrioventricular septal defect; COA, coarctation of aorta; CHD, congenital heart disease; DL, deep learning. If there were multiple models in the same study, they were extracted separately and distinguished by adding the letters “abcd” after the author; NA, not available.

### 3.2 Quality Assessment of Included Studies

**Fig. 2.**
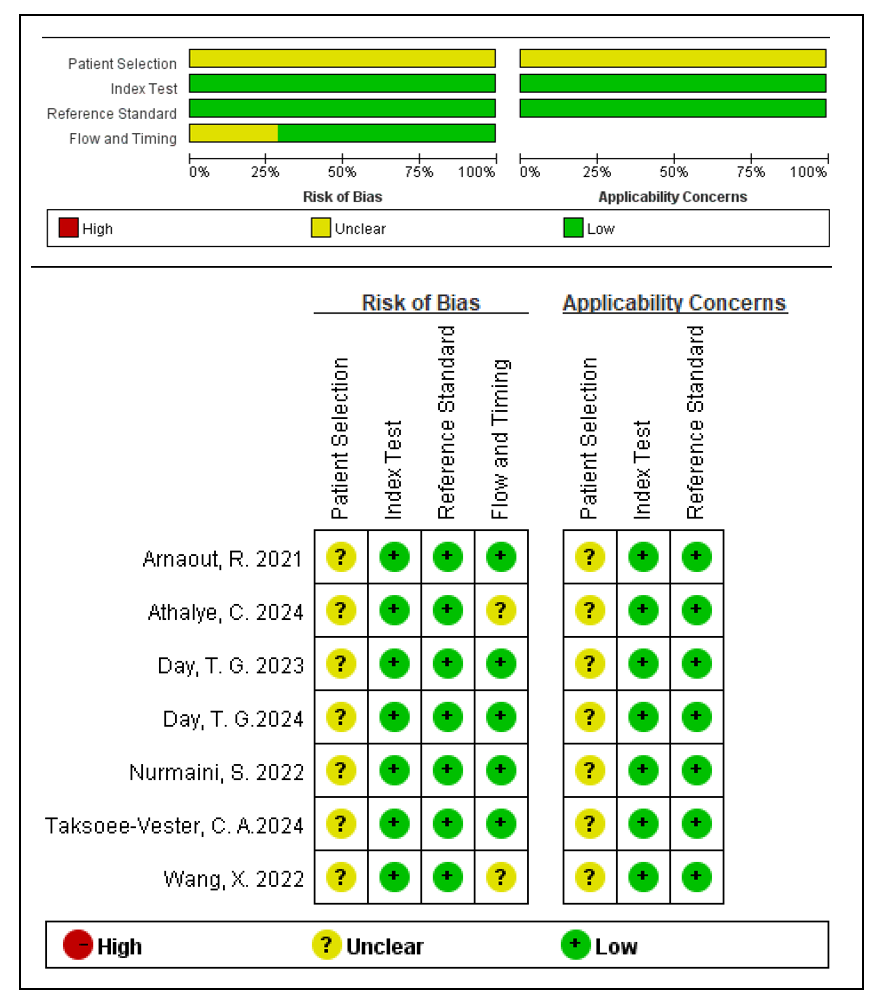
**Quality assessment using quality assessment of diagnostic accuracy studies (QUADAS-2) for included studies**.

The QUADAS-2 tool was employed for the assessment of the risk of bias. Fig. 2 displays the results of the methodological quality evaluation for the studies included in the analysis. In general, the included studies demonstrated high quality, with the majority exhibiting either a low or unclear risk of bias. In two studies, not all cases were included in the analysis due to unclear images, with one frame of images [28] and two cases [29] not included in the analysis, respectively.

### 3.3 Meta-Analysis Results of the Accuracy of AI-Assisted Diagnosis of CHD

**Fig. 3.**
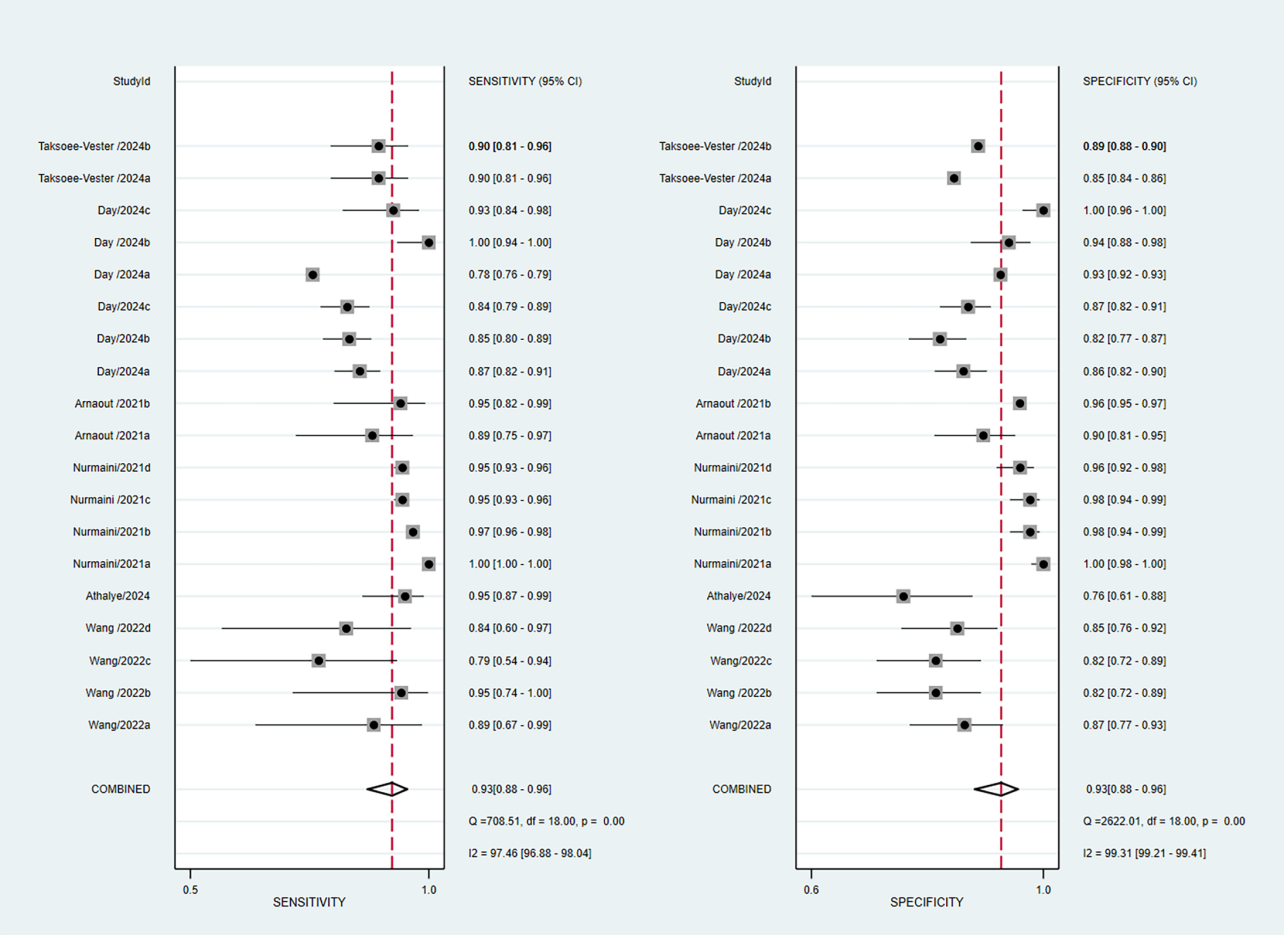
**Forest plot of combined sensitivity and specificity for the assessment of artificial intelligence (AI)-assisted diagnosis of congenital heart disease (CHD)**. CI, confidence interval.

**Fig. 4.**
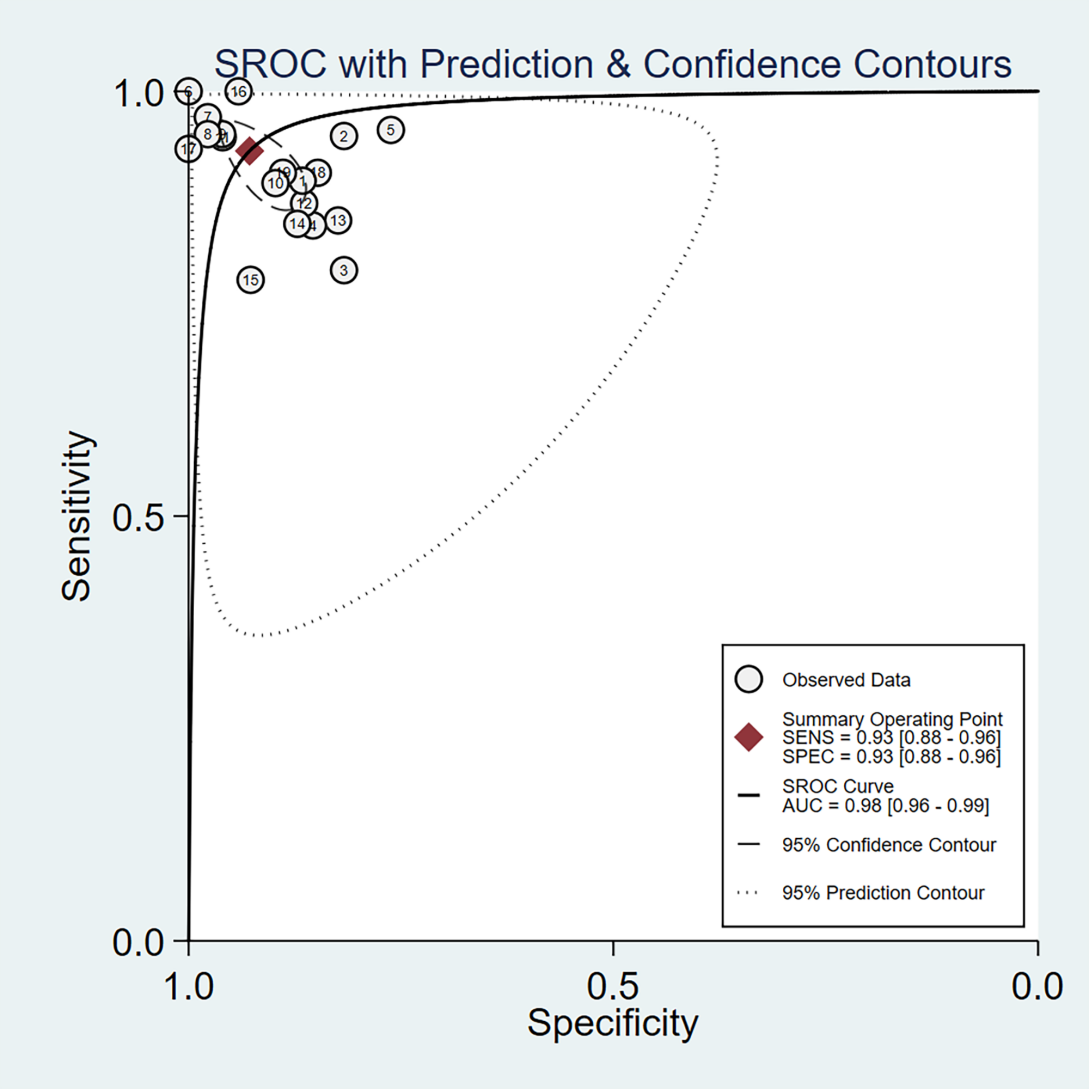
**Summary receiver operating characteristic curve of AI-assisted diagnosis of CHD**. SENS, sensitivity; SPEC, specificity; SROC, summary receiver operating characteristic; AUC, area under the curve; AI, artificial intelligence; CHD, congenital heart disease.

A total of 7 studies and 19 sets of data were included, with a pooled sensitivity of 0.93 (95% confidence interval (CI): 0.88–0.96), specificity of 0.93 (95% CI: 0.88–0.96), positive likelihood ratio of 13.0 (95% CI: 7.7–21.9), negative likelihood ratio of 0.08 (95% CI: 0.04–0.13), and diagnostic odds ratio of 171 (95% CI: 62–472). The I^2^ heterogeneity of sensitivity and specificity in this study was 97.46% and 99.31%, respectively. This heterogeneity was expected because differences in models, sample sizes, and regions among the included studies could all lead to large heterogeneity. Fig. 3 illustrates the forest plot depicting sensitivity and specificity. As demonstrated in Fig. 4, the area under the SROC curve was found to be 0.98, with a 95% confidence interval ranging from 0.96 to 0.99.

### 3.4 Publication Bias

Deek’s test was employed to assess publication bias in the included studies, yielding a value of 0.18. Since the *p* value exceeded 0.05, this implies that this meta-analysis was not affected by publication bias.

### 3.5 Subgroup Analysis

Subgroup analyses were conducted based on region and model architecture, and the results are shown in Table 2. The findings revealed that the performance of AI-aided CHD diagnosis exhibited no substantial variations across different geographic regions. The diagnostic efficacy demonstrated by these AI systems remained consistent, irrespective of the specific location or context in which they were deployed. The ResNet and DenNet architecture models had better diagnostic performance than other models, with areas under the SROC curve of 0.99 (95% CI: 0.97–0.99) and 0.92 (0.90–0.94), respectively. Since there were only three sets of data on the DenNet architecture model, it was not independently analyzed.

**Table 2.** **Subgroup analyses based on region and model architecture**.

Subgroup		Study	Sensitivity	Specificity	AUC
Total		19	0.93 (0.88, 0.96)	0.93 (0.88, 0.96)	0.98 (0.96–0.99)
Region					
	Asia	8	0.95 (0.85, 0.98)	0.96 (0.84, 0.99)	0.99 (0.97–0.99)
	America	2	0.95 (0.85, 0.98)	0.96 (0.95, 0.97)	0.99 (0.97–0.99)
	Europe	9	0.90 (0.84, 0.94)	0.90 (0.84, 0.93)	0.96 (0.93–0.97)
Models					
	ResNet/DenNet	11	0.94 (0.87, 0.98)	0.96 (0.90, 0.98)	0.99 (0.97–0.99)
	Others	8	0.91 (0.88, 0.94)	0.88 (0.82, 0.92)	0.92 (0.90–0.94)

AUC, area under the curve.

## 4. Discussion

With the development of AI, various network models have been applied to CHD image recognition in the field of deep learning, among which convolutional neural networks (CNN) [33] are representative of deep learning structure models. CNNs can autonomously extract features from raw image data and learn complex feature information, demonstrating good performance in medical image recognition and classification [26]. The models included in this study were all deep learning models, and except for one study that did not specify the model, the rest were all CNN architectures. In recent years, Xu *et al*. [34] developed a DW-NET cascaded convolutional neural network for segmenting fetal echocardiographic four-chamber view images, which can correctly locate different structures and accurately delineate the boundaries of anatomical structures, effectively extracting image indicators to assist in early prenatal diagnosis. The study tested the DW-NET on a dataset of 895 fetal four-chamber view echocardiographic images and showed that it had better segmentation performance compared to other mainstream image segmentation methods. Nurmaini *et al*. [30] compared four CNN architectures, DenseNet121, DenseNet201, ResNet50, and ResNet101, and selected the optimal CNN architecture as DenseNet201. The DenseNet 201 architecture classified seven types of CHD, including ventricular septal defect, atrial septal defect, atrioventricular septal defect, Ebstein’s anomaly, tetralogy of Fallot, transposition of the great arteries, and hypoplastic left heart syndrome, along with normal controls. The model achieved a sensitivity, specificity, and accuracy of 100%, 100%, and 100% within patients, and a sensitivity, specificity, and accuracy of 99%, 97%, and 98% between patients, respectively.

This study included a total of 7 studies and extracted 19 sets of data, with a pooled sensitivity of 0.93 and specificity of 0.93, indicating that AI models have a low rate of misdiagnosis and missed diagnosis in diagnosing CHD. The area under the SROC curve was 0.98, suggesting high accuracy of AI models in diagnosing CHD. In this study, the positive likelihood ratio was 13.0, indicating that when an AI model diagnoses CHD, the probability of diagnosing CHD is high. The negative likelihood ratio was 0.08, indicating that when an AI model diagnoses a normal heart, the probability of CHD is low. The results of this study are consistent with the study by Taksøe-Vester *et al*. [32], who used logistic regression and backward feature selection in subjects diagnosed with CoA after birth (n = 73) and healthy controls (n = 7300). The AUC of the ROC curve generated by the predictive model was 0.96, with a specificity of 88.9% and sensitivity of 90.4%.

In this study, subgroup analyses were conducted based on region and model. The subgroup analysis by Asia, Europe, and the United States showed that AI models had high diagnostic performance for CHD in different populations, with no significant differences. Most deep learning-based methods are implemented on pioneering backbone networks, the two most notable are ResNet and DenseNet, as these two architectures have a simple design strategy and good performance [35]. Layers in conventional CNN architectures are progressively linked. In contrast, the ResNet architecture employs shortcut connections, bypassing a minimum of two layers. Conversely, the DenseNet architecture offers connections originating from all feature maps in the preceding layer. This implies that all feature maps are propagated to the following layers and linked to the newly produced feature maps [30, 35]. Therefore, this study selected ResNet, DenseNet, and other models for subgroup analysis. The results showed that the area under the ROC curve for ResNet and DenseNet models in diagnosing CHD was 0.98, while the area under the ROC curve for other models was 0.92, indicating that the diagnostic performance of ResNet and DenseNet models was superior to other models.

AI application can overcome the problem of operator experience, and improve physician work flow in the analysis of fetal echocardiographic images. AI has been proven to be more reproducible and consistent than human performance [31, 36]. However, the application of AI in fetal echocardiography is still in its infancy. The database format in articles uses grayscale images or selected frames from standard videos in.avi or.mov format. Each frame needs to be labeled and unified in pixels to create a standardized data set. Currently, deep learning models used for echocardiographic diagnosis are only used to predict two-dimensional plane images. However, the information from two-dimensional planes is limited and cannot fully display the lesions. Deep learning models trained on three-dimensional ultrasound data, with ultrasound dynamic videos or spatiotemporal volumetric data of multi-view lesions, can potentially improve the diagnostic accuracy of the model while fully displaying the lesions. In addition, developing deep learning models based on multimodal ultrasound, including two-dimensional grayscale ultrasound, Doppler ultrasound, and contrast-enhanced ultrasound, can provide complementary ultrasound information and also improve the diagnostic accuracy of deep learning models [37]. Supervised learning of fetal echocardiography is most widely used in the ultrasound field, and the model training process requires big data. However, unlike photography, electrocardiogram, or chest X-ray [38, 39, 40], each ultrasound examination includes thousands of image frames. Therefore, designing a model that can handle a large number of non-independent images from datasets with relatively few individuals is an important challenge to overcome. Unsupervised learning uses unlabeled data and is used as an exploratory method. When faced with the classification of complex small-sample ultrasound data, it significantly improves the accuracy of image recognition and may become a new focus for AI application in fetal echocardiography.

This study is the latest and the first meta-analysis to investigate the value of AI-assisted diagnosis of CHD, further confirming that AI has high accuracy in diagnosing CHD, consistent with previous studies. This provides the latest evidence-based data for the clinical application of AI-assisted diagnosis for CHD. The subgroup analysis showed that the diagnostic performance of ResNet and DenseNet models was superior to other models.

This study has several limitations. First, we only retrieved literature from the English language, and important data published in non-English languages may have been missed. Due to data limitations, we were unable to conduct subgroup analyses for each model and only selected two currently more focused models and other models for subgroup analysis. We were also unable to assess their performance with larger sample sizes and greater confidence. The studies included in this study were all retrospective studies, as few prospective studies have incorporated the diagnostic accuracy of AI into clinical work. Finally, the included studies only analyzed a single congenital heart disease, or only distinguished normal heart disease from normal heart disease, and few studies distinguished fetal congenital heart disease from normal heart disease and classified specific congenital heart disease. Going forward, we plan to expand the classification of AI models to cover the entire CHD spectrum. Additional studies are needed to examine how the results of the current study apply to different fetal conditions.

## 5. Conclusions

AI has significant diagnostic value in assisting the diagnosis of CHD. ResNet and DenseNet models having the best diagnostic efficacy. In view of the small number of studies included in this meta-analysis, and the fact that they were retrospective, further multicenter studies with larger sample sizes are needed to confirm the diagnostic efficacy of AI-assisted diagnosis of CHD. With advances in AI technology, the diagnosis of fetal diseases will become increasingly accurate. We look forward to testing and improving integrated learning models in larger populations, making the expertise of fetal cardiologists available to patients in all regions, obtaining homogeneous medical resources, and applying similar techniques to other diagnoses in medical imaging.

## Availability of Data and Materials

The data used to support the findings of this study are included in the article.

## Supplementary Material

Supplementary material associated with this article can be found, in the online version, at https://doi.org/10.31083/RCM28060.
