# Supplementary material for: Artificial Intelligence-Assisted Echocardiographic Image-Analysis for the Diagnosis of Fetal Congenital Heart Disease: A Systematic Review and Meta-Analysis
Source: Rev Cardiovasc Med. 2025 Apr 27;26(4):28060. doi: 10.31083/RCM28060 (PMC12059730; doi:10.31083/RCM28060)
Supplement: Supplementary file 1 [file 2153-8174-26-4-28060-s1.zip › RCM28060-TableS1.docx]

Table S1. Search strategy

| Databases | Search strategy | Articles |
| --- | --- | --- |
| Pubmed | (((("Artificial Intelligence"[Mesh]) OR (((((((((Computational Intelligence) OR (Machine Intelligence)) OR (Computer Reasoning)) OR (AI)) OR (Computer Vision Systems)) OR (Computer Vision System)) OR (Knowledge Acquisition)) OR (Knowledge Representation)) OR (Knowledge Representations))) AND (("Diagnostic Imaging"[Mesh]) OR ((((Imaging, Diagnostic) OR (Medical Imaging)) OR (Imaging, Medical)) OR (imaging)))) AND (("Fetus"[Mesh]) OR ((((((Fetuses) OR (Fetal Structures)) OR (Fetal Structure)) OR (Mummified Fetus)) OR (Retained Fetus)) OR (fetal)))) AND (("Heart Defects, Congenital"[Mesh]) OR ((((((((Heart Abnormality) OR (Congenital Heart Defect)) OR (Malformation Of Heart)) OR (Malformation Of Hearts)) OR (Heart Abnormalities)) OR (Congenital Heart Disease)) OR (Congenital Heart Diseases)) OR (Congenital Heart Defects))) | 252 |
| Embase | ((((Artificial Intelligence) OR (((((((((Computational Intelligence) OR (Machine Intelligence)) OR (Computer Reasoning)) OR (AI)) OR (Computer Vision Systems)) OR (Computer Vision System)) OR (Knowledge Acquisition)) OR (Knowledge Representation)) OR (Knowledge Representations))) AND ((Diagnostic Imaging) OR ((((Imaging, Diagnostic) OR (Medical Imaging)) OR (Imaging, Medical)) OR (imaging)))) AND ((Fetus) OR ((((((Fetuses) OR (Fetal Structures)) OR (Fetal Structure)) OR (Mummified Fetus)) OR (Retained Fetus)) OR (fetal)))) AND ((Heart Defects, Congenital) OR ((((((((Heart Abnormality) OR (Congenital Heart Defect)) OR (Malformation Of Heart)) OR (Malformation Of Hearts)) OR (Heart Abnormalities)) OR (Congenital Heart Disease)) OR (Congenital Heart Diseases)) OR (Congenital Heart Defects))) | 33 |
| Cochrane | The same as Embase | 0 |
| Web of science | ((((Artificial Intelligence) OR (((((((((Computational Intelligence) OR (Machine Intelligence)) OR (Computer Reasoning)) OR (AI)) OR (Computer Vision Systems)) OR (Computer Vision System)) OR (Knowledge Acquisition)) OR (Knowledge Representation)) OR (Knowledge Representations))) AND ((Diagnostic Imaging) OR ((((Imaging, Diagnostic) OR (Medical Imaging)) OR (Imaging, Medical)) OR (imaging)))) AND ((Fetus) OR ((((((Fetuses) OR (Fetal Structures)) OR (Fetal Structure)) OR (Mummified Fetus)) OR (Retained Fetus)) OR (fetal)))) AND ((Heart Defects, Congenital) OR ((((((((Heart Abnormality) OR (Congenital Heart Defect)) OR (Malformation Of Heart)) OR (Malformation Of Hearts)) OR (Heart Abnormalities)) OR (Congenital Heart Disease)) OR (Congenital Heart Diseases)) OR (Congenital Heart Defects))) (Topic) | 27 |
